# Losartan counteracts the hyper-reactivity to angiotensin II and ROCK1 over-activation in aortas isolated from streptozotocin-injected diabetic rats

**DOI:** 10.1186/1475-2840-8-32

**Published:** 2009-06-22

**Authors:** Paola Failli, Chiara Alfarano, Sergio Franchi-Micheli, Edoardo Mannucci, Elisabetta Cerbai, Alessandro Mugelli, Laura Raimondi

**Affiliations:** 1 Dept. of Pharmacology, University of Florence, Viale G. Pieraccini 6, Florence, Italy; 2 Dept. of Critical Care Medicine and Surgery, Unit of Gerontology and Geriatrics, University of Florence and Azienda Ospedaliera Careggi, Florence, Italy

## Abstract

**Background:**

In streptozotocin-injected rats (STZ-rats), we previously demonstrated a role for angiotensin II (AT-II) in cardiac remodelling and insulin resistance partially counteracted by *in vivo *treatment with losartan, an AT-II receptor antagonist.

We now aimed to investigate the effect of treating diabetic STZ-rats with losartan on diabetes vascular response to vasoconstrictors.

**Methods:**

Male Wistar rats were randomly divided in four groups, two of them were assigned to receive losartan in the drinking water (20 mg/kg/day) until the experiment ending (3 weeks afterward). After 1 week, two groups, one of which receiving losartan, were injected in the tail vein with citrate buffer (normoglycemic, N and normoglycemic, losartan-treated, NL). The remaining received a single injection of streptozotocin (50 mg/kg in citrate i.v.) thus becoming diabetic (D) and diabetic losartan-treated (DL). Plasma glycaemia and blood pressure were measured in all animals before the sacrifice (15 days after diabetes induction).

In aortic strips isolated from N, NL, D and DL rats we evaluated i) the isometric concentration-dependent contractile response to phenylephrine (Phe) and to AT-II; ii) the RhoA-kinase (ROCK1) activity and expression by enzyme-immunoassay and Western blot respectively.

**Key results:**

The concentration-dependent contractile effect of Phe was similar in aortas from all groups, whereas at all concentrations tested, AT-II contraction efficacy was 2 and half and 1 and half times higher in D and DL respectively in comparison with N and NL. AT-II contracture was similarly reduced in all groups by AT-II receptor antagonists, irbesartan or irbesartan plus PD123319. HA-1077 (10 μM), an inhibitor of ROCK1 activity, reduced AT-II efficacy (Δmg/mg tissue w.w.) by -3.5 ± 1.0, -4.6 ± 1.9, -22.1 ± 2.2 and -11.4 ± 1.3 in N, NL, D and DL respectively). ROCK1 activity and expression were higher in D than in N/NL and DL aortas.

**Conclusion and implications:**

Aortas isolated from STZ-rats present hyper-contracture to AT-II mainly dependent on the up-regulation of ROCK1 expression/activity. In vivo losartan treatment partially corrects AT-II hyper-contracture, limiting the increase in ROCK1 expression/activity. These data offer a new molecular mechanism supporting the rationale for using losartan in the prevention of diabetic vascular complications.

## Introduction

Angiotensin II (AT-II), one of the effectors of the renin-angiotensin system, is among the major mediators of vascular remodelling [1]. At this site, AT-II promotes short-and long-term metabolic and functional changes, mostly by activating the type 1 receptor (AT1) located at smooth muscle cells (VSMCs). Besides being a potent contractile agent, AT-II triggers pro-inflammatory, hypertrophic [2], fibrotic and metabolic effects which include production of reactive oxygen species (ROS) [3], insulin resistance [4], extracellular matrix protein deposition [1,5-7], stimulation of cell migration and differentiation [8]. Among the intracellular signals, AT1 activation increases calcium levels and activates several kinases including the RhoA-kinase (ROCK1) pathway by recruiting its upstream activator, the small GTPase RhoA protein [1,9]. The target event of ROCK1 cascade is the phosphorylation of the myosin light chain phosphatase (MYPT1), a process that prolongs myosin light chain (MLC) activation [10,11], thus sustaining smooth muscle contraction [11,12]. Inhibition of MYPT1 by ROCK1 activation is one of the mechanisms thought to be responsible for Ca^2+ ^sensitization of smooth-muscle contraction [9,13]; even if other kinase activities, (i.e. zipper-interacting protein kinase, ZIP; integrin-linked kinase; ILK; dystrophia myotonica kinase; DMK) can inhibit MYPT1 [14-16]).

Interestingly, AT-II not only activates the RhoA/ROCK1 pathway but can also control the expression level of proteins involved in the system. Up-regulation of RhoA/ROCK1 has been described in isolated VSMCs exposed to AT-II [17,18] and in the aorta of AT-II infused rats [19,20], thus suggesting paracrine effects of AT-II on its intracellular signalling.

Increasing tissue levels of AT-II are found in experimental diabetes [21] where, together with hyperglycemia, are retained critical and initiating factors for the development of complications based on the so-called "vascular dysfunction" (endothelial and smooth muscle dysfunction), a condition modifying the function (hyper response to vasoconstrictors) and the metabolism (onset of insulin resistance and increase of oxidative stress) of the vascular bed. Up-regulation of ROCK1 activity has been demonstrated in the vasculature of insulin-resistant animals independently of the experimental model studied [22,23] whereas hyperglycemia "*per se" *increases ROCK1 activity in isolated vascular cells [24]. Therefore, high AT-II and hyperglycemia, may synergistically increase the activity of the biochemical machinery functionally coupled to muscle contraction. This implies that AT-II and hyperglycemia might play a determinant role in priming diabetes VSMCs dysfunction.

In streptozotocin-injected rats (STZ-rats), a widely used experimental model for the study of diabetes-related cardiovascular complications, the extent of the vascular dysfunction depends on the duration of the pathology [25].

We have previously reported that STZ-rats, 2 weeks after injection, present typical diabetes-related cardiac electrophysiological remodelling and insulin resistance [26]. Interestingly, *in vivo *treatment of diabetic rats with losartan, an antagonist of AT- II type 1 receptors, prevented both the electrophysiological and the metabolic alterations, without affecting hyperglycemia. These results confirmed that selective AT1 antagonists represent useful tools for investigating AT-II roles in priming diabetes complications.

In the present study we aimed to extend our previous observations investigating whether aortas from "early" diabetic rats, show hyper-response to vasoconstrictors including Phe and AT-II and the molecular mechanism underlying this effect. To this aim we studied the functional response to Phe and AT-II in strips prepared from aortas isolated from 2-week STZ-rats "*in vivo*" treated and not treated with losartan (20 mg/kg/day^-1^). By using this pharmacological strategy, we aimed to investigate the role of AT-II type 1 receptor in the development of vascular hyperreactivity dependent on hyperglycemia. In this respect, other approaches such the use of angiotensin converting enzyme inhibitors (ACE-I) could be misleading, since these molecules can influence other important vascular regulatory pathways independently from their inhibitory effect on AT-II formation.

We demonstrate that an *in vivo *losartan treatment reduces vascular hyper-reactivity to AT-II, that according to our data, is mainly dependent on an increase in the RhoA/ROCK1 pathway.

## Methods

All of the experiments were carried out in accordance with the European Communities Council Directive of 24 November, 1986 (86/609/EEC) for experimental animal care.

Wistar rats aged 12–14 weeks (Charles River, Calco [LC], Italy) were randomly divided into four groups (see below for description) and allowed free access to standard dried chow diet and water whose consumption was monitored daily.

Two groups of rats were assigned to receive losartan in the drinking water (20 mg/kg/day) until the end of the experiment (3 weeks afterward). After 1 week, two groups, one of the two receiving losartan were injected in the tail vein with citrate buffer pH 4.5 (normoglycemic, N and normoglycemic, losartan-treated, NL). The other two groups received a single injection of STZ (50 mg/kg in citrate buffer pH 4.5). According to our previous results [26] we checked plasma glycaemia of rats 48 h following STZ injection and only those rats whose glycaemia was higher than 14 mM were considered diabetic and included in our schedule of treatment (diabetic, D and diabetic, losartan-treated, DL, a total of 40 rats). The losartan concentration was adjusted according to body weight and water consumption to maintain a dosage of 20 mg/kg/day [26] and the treatment was interrupted 24 h before sacrificing.

Animals were put in metabolic cages and fasted overnight with free access to water before the sacrifice.

### Blood pressure measurement

Diastolic and systolic blood pressure were measured the day before sacrifice in conscious rats using BP-2000 (Visitech Systems, Apex, USA) a non-invasive computerised system for recording blood pressure from tails of rodents.

### Functional studies on rat thoracic aortas

The thoracic aorta was rapidly dissected out and placed in ice-cold, oxygenated (5% CO_2 _in O_2_) Krebs-Henseleit solution (K-H) of the following composition (mM): 110 NaCl, 25 NaHCO_3_, 4.8 KCl, 1.2 KH_2_PO_4_, 1.2 MgSO_4_, 11 D(+)glucose, 2.5 CaCl_2_. It was then cleaned of loosely adhering fat and connective tissue, cut into helical strips, 2 mm in width and 20 mm in length, and placed in an organ bath of 2 ml volume [27]. Then, strips were incubated with pre-warmed, oxygenated K-H (37°C for at least 60 min), the resting tension being set at 0.7 g. After equilibration, cumulative concentration-response curves of phenylephrine (Phe) were performed. At the sub-maximal Phe concentration of 100 nM, the relaxing concentration-dependent effect of acetylcholine was tested [28]. After washings, AT-II concentration-response curves were performed on 100 nM Phe pre-contracted strips. The pharmacological modulation of AT-II contracture was investigated in aortic strips perfused for 30 min at 37°C with 1 μM irbesartan or 1 μM irbesartan plus 1 μM PD123319 (type 1 and type 2 AT-II receptor antagonists respectively) or 10 μM HA-1077, an inhibitor of type 1 RhoA kinase (ROCK1) activity [29].

At the end of the experiments, strips were rinsed with K-H solution for 10 min at 37°C, removed from apparatus, blotted on filter paper and weighted. Contraction was expressed either as mg of contractile force for mg of wet tissue weight (mg/mg tissue w.w.) or as a percentage of 100 nM Phe contracture (100%) as indicated.

Acetylcholine, AT-II, HA-1077, PD123319 and Phe were obtained from Sigma-Aldrich, St. Louis, MO, USA. All other reagents were of analytical grade.

### ROCK1 expression level in rat aorta homogenates

The abdominal aorta from rats belonging to the different groups was isolated, cleaned and washed in cold saline solution (0.9% NaCl), and frozen in liquid nitrogen. Aortic samples were rinsed, weighed and homogenized (1 mg/ml) in lysis buffer of the following composition (in mM): 50 Tris·HCl pH 7.5, 1 EDTA, 150 NaCl, 1 Na_3_VO_4_, 10 NaF and complete protease inhibitor cocktail tablet (Sigma-Aldrich, St.Louis, MO, USA. The homogenate was centrifuged (1,000 × *g *× 10 min at 4°C) to remove cell debris. The resulting supernatant was frozen at -80°C for later analysis by SDS-PAGE and immunoblotting. The protein concentration of the crude lysate was determined by the BCA protein assay reagent kit (Pierce, ROCK1eford, IL, USA). Proteins separated on a 4–12% (w/vol) acrylamide gel (Nu-Page Gel, Invitrogen, Milan, Italy) were transferred to polyvinylidene difluoride membranes and probed with mouse anti-rabbit ROCK1 polyclonal antibodies (1:200 dilution; St. Cruz Biotechnology Inc., CA, USA) or with polyclonal anti-rabbit actin (1: 1000 dilution; Sigma-Aldrich, St. Louis, MO, USA) overnight at 4°C.

After extensive washings, a goat anti-rabbit peroxidase conjugated antibody was added (1:2000 dilution; Sigma-Aldrich, St. Louis, MO, USA) and immunodetected bands were visualized by ECL. Densitometric analysis of autoradiographic bands were referred to actin expression taking into account the size and the area of the band (Scion software Image Corp).

### Determination of ROCK1 Activity

Aortas were rinsed, weighed, frozen in liquid nitrogen and homogenized (3 mg/ml) in lysis buffer (see above for composition). Homogenates were then centrifuged (30.000 × g × 30 min) and the resulting supernatant was used as enzymatic source of ROCK1.

Kinase activities were estimated in each sample by a non isotopic ELISA test (Cyclex Co, Ltd; Nagano, Japan), according to the manufacturer's instructions. For each sample, the optimal dilution (from 1:5 to 1:40) was established to obtain a maximal optical density (OD). Thereafter, each supernatant was pre-incubated for 30 min in the absence (total kinase activities, e. i. zipper interacting protein kinase, ZIP, dystrophia myotonica protein kinase DMK, integrin-linked kinase, ILK and ROCK1) or in the presence with either 10 μM HA-1077 or 1 μM Y-27632 [30] purchased by Sigma-Aldrich, St. Louis, MO, USA), two inhibitors of ROCK1 kinase activity. The difference between the OD at 450 nm measured in the absence and in the presence of ROCK1 inhibitors was calculated and referred to as ROCK1 activity. Each sample was assayed in duplicate.

Results are expressed as Arbitrary Units (A.U.) i.e. the OD at 450 nm normalized to the protein content of each original supernatant.

### Statistical methods

Values are presented as the mean ± SEM and analysed with one-way ANOVA followed by Bonferroni's t test. Effective concentrations at 20%, 50% and 80% of the maximum response (EC_20_, EC_50 _and EC_80 _respectively) for each AT-II concentration response curve was calculated using Origin Microcal^® ^(version 7) program by extrapolating experimental points with a sigmoidal curve. A P < 0.05 was considered as significant.

## Results

### Characterization of animal groups

Two weeks after STZ (50 mg/kg) injection, rats from groups D and DL showed a significant increase in water consumption, plasma glycemia and a marked reduction in body and heart weight vs. citrate-injected rats (group N and NL). Therefore, animals from groups D and DL were considered diabetic. No change in the heart weight-to-body weight ratio, an index of cardiac hypertrophy was observed (Table 1). Losartan treatment did not modify any of the parameters observed either in NL or in DL group.

**Table 1:** Metabolic characteristics of normoglycemic (group N), normoglycemic, *in vivo *losartan treated (group NL), diabetic (group D) and diabetic, *in vivo *losartan treated (group DL) rats.

**Groups**	**Plasma Glycemia** **(mM)**	**Daily Water Consumption (ml)**	**Daily Food Intake** **(g)**	**Body Weight** **(g)**	**Heart Wet Weight** **(mg)**	**Heart Weight/Body Weight** **(mg/g)**
**N (n = 7)**	7.0 ± 0.014	29.9 ± 2.2	17.9 ± 0.8	262.1 ± 10.1	993.8 ± 37	3.8 ± 0.2
**NL (n = 7)**	9.0 ± 0.033	29.3 ± 1.6	19.4 ± 0.5	254.7 ± 9.1	951.3 ± 36	3.7 ± 0.1
**D (n = 14)**	20.0 ± 0.18*	133.8 ± 6.8*	28.6 ± 0.9*	217.6 ± 5.4*	806.5 ± 33^	3.7 ± 0.1
**DL (n = 12)**	18.7 ± 0.25#	130.1 ± 8.6#	27.3 ± 1.4#	207.5 ± 10.2#	851.5 ± 60	4.1 ± 0.4

Values presented are the mean ± SEM of the indicated number of rats (n). P < 0.001 vs. N; **# **P < 0.001 vs. NL; ^P < 0.01 vs. N. Wistar rats aged 12–14 weeks were randomly assigned to the different groups.

### Blood pressure levels in the different groups of animals

As shown in Table 2, the animals belonging to the different groups had similar BP values irrespective of the presence of diabetes. Moreover, losartan treatment did not modify BP either in NL or in DL groups.

**Table 2:** Blood pressure levels and heart rate of normoglycemic (group N), normoglycemic, *in vivo *losartan treated (group NL), diabetic (group D) and diabetic, *in vivo *losartan treated (group DL) rats.

	**Blood Pressure** (mmHg)		**Heart Rate** **(beats/min)**
**Groups**	**Systolic**	**Diastolic**	

**N**	139.2 ± 16.8	110.6 ± 2.9	393.6 ± 10.4
**NL**	144.0 ± 4.4	112.4 ± 5.5	385.4 ± 8.2
**D**	145.0 ± 8.1	119.9 ± 10.9	382.2 ± 10.3
**DL**	139.7 ± 6.0	99.4 ± 9.6	370.7 ± 4.2

Values are the mean ± SEM of 6 animals.

### The effect of phenylephrine and acetylcholine in all animal groups

**Figure 1:**
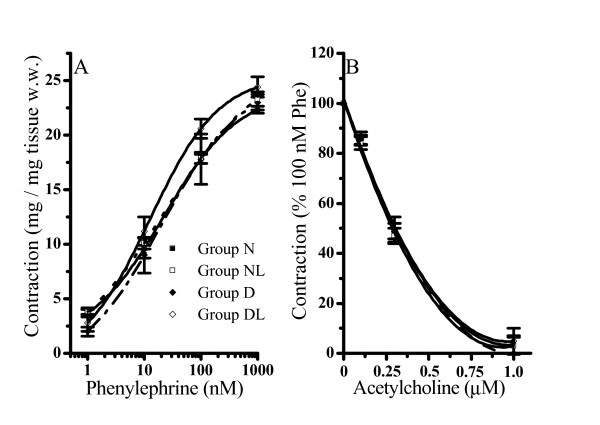
**Effect of phenylephrine (panel A) and acetylcholine (panel B) on aortic strips isolated from normoglycemic (group N), normoglycemic *in vivo *losartan treated (group NL), diabetic (group D) and diabetic *in vivo *losartan treated (group DL) rats**. Cumulative concentration-response curves of agonists were performed on thoracic aortic strips stretched to 0.7 g resting tension with an endothelial-intact layer. Values are the mean ± SEM of at least 4 experiments. Panel A) phenylephrine contraction. Panel B) acethylcholine effect was evaluated on 100 nM phenylephryne (Phe) pre-contracted thoracic aortic strips (100% of contraction).

Aortas isolated from rats belonging to the different groups showed a similar contractile response to Phe. As illustrated in Figure 1 (panel A), cumulative concentration-response curves of Phe were superimposable in the full range of concentrations tested (1–1000 nM) and it reached its maximal response at 1000 nM in all groups.

In aortas pre-contracted with the sub-maximal Phe concentration of 100 nM, acetylcholine (from 0.1 to 1 μM) induced a similar extent of relaxation, suggesting that all the preparations conserved a functionally active endothelial layer (Figure 1B).

### The contractile effect of angiotensin II in N (normoglycemic) and D (diabetic) groups

**Figure 2:**
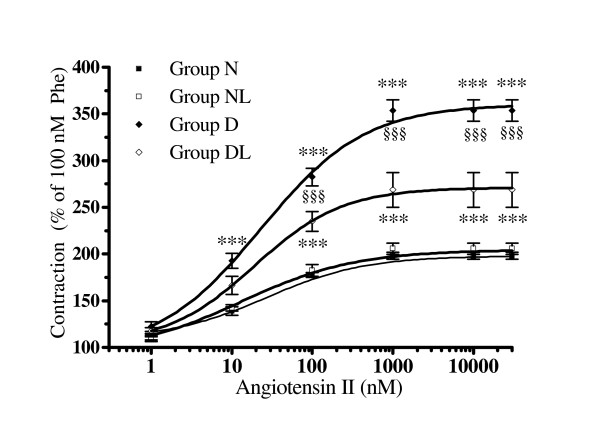
**The effect of angiotensin-II in aortic strips isolated from normoglycemic (group N), normoglycemic *in vivo *losartan treated (group NL), diabetic (group D) and *in vivo *losartan treated diabetic rats (group DL)**. Cumulative concentration-response curves of angiotensin II (bolus) were performed on 100 nM phenylephryne (Phe) pre-contracted thoracic aortic strips stretched to 0.7 g resting tension with an endothelial-intact layer. Values are the mean ± SEM of 4 experiments. ***P < 0.001 vs. N and NL; ^§§§^P < 0.001 vs. DL

AT-II concentration-response curves were performed on 100 nM Phe pre-contracted preparations. AT-II further increased the contraction in aortas isolated from each experimental animal group (Figure 2). In particular, the AT-II contracture was higher in D than in N group in the concentration range from 10 to 30.000 nM (P < 0.001) within the experimental groups reaching at the maximal concentration, an effect that was 2.5 times higher in group D than in aortas from group N. Notwithstanding these differences in contraction efficacy, similar values of EC_20_, EC_50 _and EC_80 _were calculated for AT-II (Table 3).

**Table 3:** Effective concentration (EC, nM) at 20%, 50% and 80% of maximum angiotensin II contracture in aortas isolated from normoglycemic (group N), *in vivo *losartan treated normoglycemic (group NL), diabetic (group D), and *in vivo *losartan treated diabetic rats (group DL) in control condition and in the presence of irbesartan 1 μM and 1 μM irbesartan plus 1 μM PD123319.

EC 20% (nM)			
N	3.4 ± 0.77	3.2 ± 0.67	NC
NL	3.3 ± 0.50	4.7 ± 0.72	NC
D	4.4 ± 0.81	4.7 ± 1.01	3.5 ± 0.86
DL	3.6 ± 0.70	4.0 ± 0.88	4.1 ± 0.90

EC 50% (nM)			

N	22.4 ± 3.88	22.1 ± 3.47	NC
NL	19.8 ± 1.16	28.1 ± 9.79	NC
D	19.6 ± 2.22	18.3 ± 2.49	23.2 ± 3.06
DL	22.7 ± 2.10	25.9 ± 4.85	18.3 ± 2.14

EC 80% (nM)			

N	106.6 ± 19.64	121.9 ± 21.30	263.8 ± 68.43
NL	117.8 ± 27.60	128.1 ± 5475	170.7 ± 11.02
D	118.4 ± 39.79	117.8 ± 30.41	215.5 ± 81.48
DL	98.5 ± 29.96	160.1 ± 18.72	277.6 ± 99.14

From each curve, the EC_20_%. EC_50_% and EC_80_% were calculated by fitting experimental points with a sigmoidal extrapolation. Values are the mean ± SEM of at least 4 fittings for each experimental group. NC = not calculated

### The contractile effect of angiotensin -II in normoglycemic and diabetic rats treated with losartan (groups NL and DL)

The AT-II concentration-response curve measured in aortas from group NL was comparable to that obtained in the N group (Figure 2), indicating that *in vivo *losartan treatment did not affect the *in vitro *AT-II response, thus excluding interference by losartan pharmacokinetic on AT-II contraction.

On the contrary, in DL aortas, AT-II contractile effect was lower than that measured in aortas from D group in the range from 100 to 30.000 nM (P < 0.001, Figure 2). At this latter concentration, the AT-II contractile effect was significantly decreased from +353.3% of group D to +268.8% in group DL (P < 0.001 vs. D), a value which remained higher than that measured in groups N and NL (P < 0.001).

The analysis of concentration-response curves indicates that the potency (nM) of AT-II contraction at 20, 50 and 80% of its maximum effect did not change significantly (P > 0.05) among the experimental groups (Table 3), whereas the contractile efficacy of AT-II (intrinsic activity) was drastically increased in D and to a less extent in DL.

### Identification of angiotensin-II receptor subtypes involved in the AT-II contraction

To evaluate the involvement of AT1 and type 2 receptors (AT2) on AT-II effect, rat aortas pre-contracted with Phe were incubated for 30 min at 37°C in the presence of irbesartan or irbesartan plus PD123319 (both at a concentration of 1 μM) before performing AT-II cumulative concentration-response curves.

**Figure 3:**
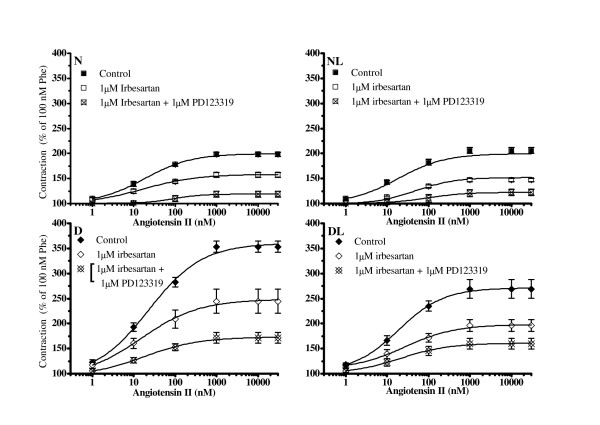
**The effect of irbesartan and irbesartan plus PD123319 on angiotensin-II induced contraction in aortic strips isolated from normoglycemic (N), *in vivo *losartan treated normoglycemic (NL), diabetic (D) and *in vivo *losartan treated diabetic rats (DL)**. Cumulative concentration-response curves of angiotensin-II (bolus) were performed on 100 nM phenylephrine (Phe) pre-contracted thoracic aortic strips stretched at 0.7 g resting tension with an endothelial-intact layer and pre-incubated with 1 μM irbesartan or 1 μM irbesartan plus 1 μM PD123319. The contraction obtained with 100 nM Phe was set as 100%. Values are the mean ± SEM of 4 experiments. Concentration-response curves of angiotensin II were significantly (at least P < 0.05) reduced by irbesartan in aortas from all groups. At condition of irbesartan plus PD123319, angiotensin-II contraction was reduced further (at least P < 0.05). For the clarity, significant level marks are omitted from the figure.

As shown in Figure 3, in the presence of irbesartan, the AT-II maximal contracture was reduced by about 50% in all groups.

The contemporaneous presence of both receptor antagonists, irbesartan plus PD123319, did not completely abolish the AT-II contracture. In fact, a residual contraction estimated as 18.9 ± 6.17 in N, 20.2 ± 2.75% in NL, 28.9% ± 4.4 in D and 34.6 ± 4.99% in DL was observed (P > 0.05 not significant; Figure 3). Notwithstanding this, AT-II potency (nM) at 20, 50 and 80% of its maximum effect was not significantly different from normo (N and NL) to hyperglycaemic (D and DL) aortas (Table 3).

Interestingly, the pre-incubation of aortas with PD123319 in the absence of irbesartan did not produce neither a reduction or an increase of AT-II maximum effect.

### Angiotensin-II hyper-contracture in diabetic rat aorta is reduced by a ROCK1 inhibitor

All the evidence collected so far suggested that the AT-II-augmented contraction observed in aortas from groups D and DL could depend on an enhancement of some intracellular transduction pathways. Among the possible targets we explored the involvement of the RhoA/ROCK1 signalling pathway. To this aim, cumulative concentration-response curves of Phe and then of AT-II were carried out in aortas pre-incubated (30 min) in the absence or in the presence of 10 μM HA-1077, a concentration currently used in *in vitro *studies [29,31] to selectively inhibit the ROCK1 activity.

**Figure 4:**
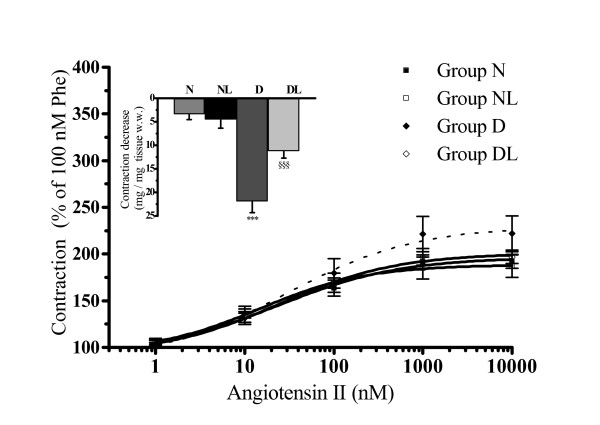
**The effect of HA-1077 on angiotensin-II induced contraction in aortic strips isolated from normoglycemic (group N), *in vivo *losartan treated normoglycemic (group NL), diabetic (group D) and *in vivo *losartan treated diabetic rats (group DL)**. Cumulative concentration-response curves of angiotensin-II (bolus) were performed on 100 nM phenylephrine (Phe) pre-contracted thoracic aortic strips stretched to 0.7 g resting tension with an endothelial-intact layer and pre-incubated with 10 μM HA-1077. The contraction obtained with 100 nM Phe was set as 100%. The insert shows the decrease in contraction (reported as mg/mg of tissue w.w., absolute value) obtained with HA-1077 on angiotensin-II-induced (1 μM) contracture. Values are the mean ± SEM of 4 experiments. ***P < 0.001 vs. all other groups; ^§§§^P < 0.01 vs. N and D groups.

The presence of HA-1077 did not affect the contraction produced by Phe which remained similar in N, NL or D and DL groups. On the contrary, HA-1077 significantly reduced the contracture of AT-II in D and DL at all the concentrations tested, while it did not affect the AT-II concentration-response curves in N and NL aortas. As shown in Figure 4 (insert) at the highest concentration (1000 nM), AT-II contraction was reduced (Δ mg mg^-1 ^tissue w. w.) by -3.5 ± 1.07 in N (n = 4), -4.6 ± 1.75 in NL (n = 4), -22.1 ± 2.25* in D (n = 4), and -11.4 ± 1.32^§ ^in DL (n = 4; Figure 4, (insert); *P < 0.05 vs. N, NL and DL; ^§^P < 0.05 vs. N and D). It is worth noting that in the presence of HA-1077, the AT-II concentration-response curves in group D and DL become indistinguishable from those obtained in groups N and NL (Figure 4).

### Losartan treatment reduced ROCK1 enzyme activity

To further investigate the role of ROCK1 in diabetic aorta hypercontractility, we measured the MYPT1 phosphorylation, the major target of ROCK1 cascade, as indicative of ROCK1 phosphorylation activity. Since MYPT1 phosphorylation can be performed by several kinases, ROCK1 involvement was calculated by running experiments in the absence and in the presence of two ROCK1 specific inhibitors: HA-1077 (10 μM) and Y-2763 (1 μM). The difference between the total MYPT1 phosphorylation and residual (ROCK1 inhibitor insensitive) levels, provided an estimation of the ROCK1 activity

Our result show that MYPT1 phosphorylation level was higher in D aortas in comparison with those in N, NL and DL. When ROCK1 inhibitors (HA-1077 and Y-27632) were used, MYPT1 phosphorilation was significantly reduced only in D (Table 4) but not in N, NL and DL.

**Table 4:** Kinase-dependent MYPT1 phosphorylation (phospho-treonine) in aortic samples from normoglycemic (group N), normoglycemic *in vivo *losartan treated (group NL), diabetic (group D) and diabetic *in vivo *losartan treated (group DL) rats.

**MYPT1 phosphorylation (Arbitrary Units)**			
**Groups**	**No drug addition**	**+HA-1077**	**+ Y-27632**

**N**	2.02 ± 0.27	1.19 ± 0.10 (Δ: 0.83 ± 0.18)	0.72 ± 0.068 (Δ: 1.3 ± 0.136)
**NL**	1.99 ± 0.135	0.99 ± 0.05 (Δ: 1.0 ± 0.3)	0.56 ± 0.03 (Δ: 1.43 ± 0.29)
**D**	3.4 ± 0.18***^,§§^	0.47 ± 0.109 (Δ:2.92 ± 0.28***;§§	0.72 ± 0.04 (Δ: 2.67 ± 0.25*)
**DL**	2.17 ± 0.083	0.52 ± 0.171 (Δ: 1.65 ± 0. 24)	0.63 ± 0.039 (Δ: 1.55 ± 0.11)

***P < 0.001 vs. N and NL; §§P < 0.01 vs. DL; *P < 0.05 vs. N, NL and DL. Samples from rat aortas were prepared as described in "Methods". Values represent the mean ± SEM of 4 rat aortas from each group.

Δ = difference between the MYPT1 phosphorylation measured in the absence of drug addition and that obtained in the presence of indicated ROCK1 inhibitors.

Interestingly enough, the extent of the inhibitor insensitive MYPT1 phosphorylation gained similar values in all groups, thus suggesting that ROCK1 activity was a predominant mechanism for MYPT1 phosphorilation in D aortas (Table 4).

### ROCK1 protein expression: the effect of losartan

**Figure 5:**
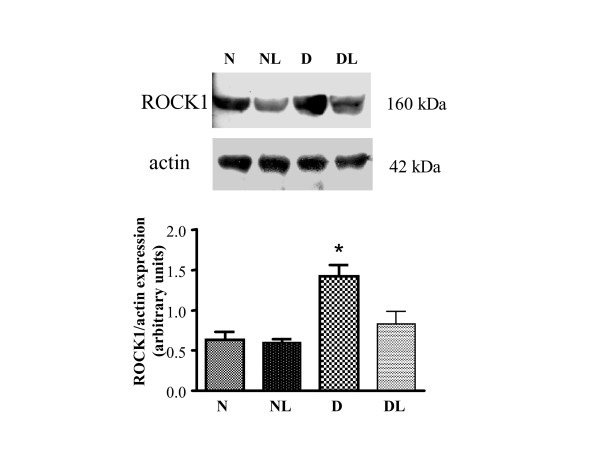
**ROCK1 expression in aorta homogenates from normoglycemic (group N), *in vivo *losartan-treated normoglycemic (group NL), diabetic (group D) and *in vivo *losartan treated diabetic rats (group DL)**. Upper panel: ROCK1 protein expression level evaluated by Western-blot (a representative experiment is shown). Lower panel: densitometric analysis of bands normalized to actin expression. Three gels were analyzed and results represent the mean ± SEM of 3 experiments. *P < 0.05 vs. all other groups.

To investigate whether over-activation of ROCK1 might be the consequence of ROCK1 over-expression, we performed a Western-blot analysis of aortic proteins. Our results, presented in Figure 5 (upper panel), show that the level of ROCK1 expression were indeed higher in aortas from D when compared to that of N and NL (P < 0.05 vs. N and NL, figure 5 bottom panel). Furthermore, as for the functional data, the expression of ROCK1 lowered in consequence of losartan treatment. In fact, in DL aortas, the expression of ROCK1 was similar to that found in N and NL aortas (Figure 5, bottom panel).

## Discussion

Our data demonstrate that aortas isolated from 2-week STZ rats present hyper-contracture to AT-II, but not to the alpha1-agonist Phe, and that this hyper-response is partially prevented by an *in vivo *treatment with losartan. In fact, in diabetic aortas, AT-II efficacy of contracture was higher (hyper-contracture) than that in aortas from normoglycemic rats and *in vivo *losartan treated diabetic rats.

The effect of AT-II in diabetic aortas, occurs at conditions of conserved vasorelaxant capacity to acetylcholine, in line with data obtained in a similar early model of STZ-rats [25], and in the absence of any significant change in systemic pressure (Table 2) suggesting that endothelial dysfunction might not be functionally measurable at our experimental settings.

Analyzing the concentration-response curves, we concluded that the augmented AT-II intrinsic activity found in diabetic aortas was not the consequence of increasing AT-II receptor levels. In fact, pharmacological evidence indicated that in aortas from all the groups of rats: 1) AT-II maintained a similar potency at 20, 50 and 80% of its maximum effect in determining contracture; 2) irbesartan, as well as irbesartan plus PD123319, reduced analogously AT-II contracture. These results confirmed that the contribution of AT1 and AT2 in determining AT-II hyper-contracture was similar in aortas from normo and hyperglycemic rats. To note, a residual, AT1 and AT2 antagonist-insensitive contracture, whose extent was again similar in normo and hyperglycemic aortas, was measurable. This residual contracture, might result from the release of some contractile factor(s) induced by AT-II or from the presence of an AT-II receptor with a low susceptibility to irbesartan and PD123319 both used at selective and effective concentrations [32,33].

From all these data, we concluded that the increased response to AT-II in diabetic aortas could be explained by an over-activation of some transduction system(s) coupled to AT-II receptors.

Being the hyper-contracture to AT-II reduced in DL group, we hypothesised that the *in vivo *AT1 receptor activation might play a fundamental role in determining AT-II effect in diabetic aortas.

Since the ROCK1 system is among the effectors of the AT1 cascade, we explored its involvement in AT-II hyper-contracture. To this aim we verified the AT-II effect in N, NL, D and DL aortas exposed to HA-1077. Our result showed that HA-1077 did not affect AT-II contracture in normoglycemic aortas (N and NL groups), whereas it strongly reduced the AT-II contraction in D and DL groups (-22.1 ± 2.25 and -11.4 ± 1.32 mg/mg tissue w. w. respectively at the 1 μM AT-II). In the presence of HA-1077, diabetic aortas responded to AT-II similarly to normoglycemic animals. Increased ROCK1 signalling, in terms of protein expression and enzyme activity, was also demonstrated in diabetic aortas where ROCK1 protein expression and its activity were two and around 2–3 times higher respectively than that measured in N and NL rats. These results showed quite a good correlation between ROCK1 protein expression/activity with functional data.

It is also noteworthy that, among the kinases able to phosphorylate MPT/MYPT1, only ROCK1 activity was up-regulated in our diabetic aortas.

Interestingly, ROCK1 expression and activity were significantly lower in DL than in D aortas. This allowed us to conclude that in vivo losartan treatment corrected diabetic AT-II hyper-contracture, preventing the increase in ROCK1 expression and activity. The association between AT-II hyper-response and ROCK1 was corroborated by the evidence that HA-1077 did not modify Phe contractility, likely for the different Gprotein subtypes activated by the two agonists. Indeed, RhoA/ROCK1 activation is mainly dependent on G12/13 protein-linked AT1 [1], while other G proteins are less effective in this pathway. Other authors have reported a low involvement of RhoA/ROCK1 pathway in alpha adrenergic stimulation [22,34]. Indeed, alpha1-contracture was unchanged in our experimental groups as already described elsewhere [25].

Accumulating evidence spotlight on RhoA/ROCK1 signalling as a critical regulator in determining Ca^2+ ^sensitization of the vascular smooth muscle cells contractile machinery [10,22,30-36]. In line with this, ROCK1 has attracted significant interest as a potential target for the treatment of a wide range of pathological conditions including cancer, neuronal degeneration, kidney failure, asthma, glaucoma, osteoporosis, erectile dysfunction, insulin resistance and its cardiovascular complications [37]. Despite the considerable interest and the development of potent ROCK1 inhibitors, there is little information on clinical trials with selective ROCK1 inhibitors [37]. On the contrary, numerous clinical trials have extensively confirmed the efficacy and safety of losartan and other AT1 blockers in diabetic and or hypertensive patients [38,39].

Although the STZ-rat is one among the experimental models of diabetes, it shows suitability for studying the basic mechanisms of diabetic cardiovascular complications and their time-dependent progression [25]. Thus, our data obtained on a model of "early diabetes", could suggest a new molecular mechanism supporting the protective role of losartan in diabetic vascular complications.

## Abbreviations

AT-II: Angiotensin II; D: Diabetic rats; DL: Diabetic: losartan treated rats; EC_20_, EC_50 _and EC_80_: effective concentration at 20, 50 and 80% of maximum response; MYPT1: myosin light chain phosphatase; N: Normoglycemic rats; NL: Normoglycemic, losartan treated rats; Phe: Phenylephrine; ROCK1: RhoA-kinase; STZ: Streptozotocin;Y-27632,(+)-(*R*)-trans-4-(1-aminoethyl)-*N*-(4-pyridyl) cyclohexanecarboxamide dihydro-chloride monohydrate; VSMCs: vascular smooth muscle cells; w.w.: wet weight.

## Competing interests

The authors declare that they have no competing interests.

## Authors' contributions

PF carried out experimental design, elaboration and statistical analysis of functional data on Phe and AT-II contractile effect; SFM: carried out the functional experiments on Phe and AT-II contractile effect; CA: carried out Western-blot analysis for ROCK1 expression and determination of ROCK1 enzyme activity EM: intellectual support for writing the paper as an expert in diabetology; AM: senior scientist of the group, general and intellectual support; EC: intellectual and general support as an expert in experimental cardiology; LR: experimental design of biochemical and molecular data, elaboration and preparation of the manuscript together with PF. All authors have read and approved the final manuscript.
